# Root causes of deaths by suicide amongst patients under the care of a mental health trust: a thematic analysis

**DOI:** 10.1192/bjo.2021.819

**Published:** 2021-06-18

**Authors:** Dhruba Bagchi, Kerry Webb, Opeyemi Odejimi

**Affiliations:** 1 Birmingham Community Healthcare NHS Foundation Trust; 2 Birmingham and Solihull Mental Health NHS Foundation Trust

## Abstract

**Aims:**

This study explores common themes emerging from root causes of Serious Incident (SI) reports of mental health service users who died by suicide under the care of a mental health trust.

**Background:**

Suicide is a global health problem. It is estimated every year about 800,000 people die by suicide worldwide. Previously, the United Kingdom (UK) reported a significant reduction. However, the latest report in 2018 indicated a marked increase. Furthermore, 28% of people who died by suicide in the UK were under the care of mental health service 12 months prior to their death. The causes of suicide are not usually straightforward, but sometimes could be preventable. Thus exploring the root causes is a step in the right direction to preventing this global problem.

**Method:**

Thematic analysis was carried to identify themes emerging from the Root Causes (RCs) within the Serious Incident (SI) reports of patients who died by suicide while under the care of the Trust between January 1st, 2017 and July 31st, 2018. Over the 18 month period, there were 71 deaths, of which 36 were ruled as suicide by the coroner. A further 16 were considered by the review team as possible suicide and were therefore included to increase the scope of learning. This review is therefore based on 48 cases.

**Result:**

Three main themes emerged from this study. They are patient, professional and organisational factors. Majority of the death were patient related factors, particularly exacerbation of patient's mental health condition. Furthermore, the most frequently occurring professional and organisational factor were issues around patient risk assessment and management and inadequate psychiatric bed respectively.

**Conclusion:**

The findings of this study have helped gained an understanding of the perceived causes of death of patient who died by suicide. It is hoped that this will in turn influence the manner in which, decisions, policies and resource allocation are carried out to further prevent and reduce the incidence of suicide, particularly amongst mental health patients.

